# Acupuncture for painful diabetic peripheral neuropathy: a systematic review and meta-analysis

**DOI:** 10.3389/fneur.2023.1281485

**Published:** 2023-11-16

**Authors:** Luolin Zhou, Tong Wu, Zhishan Zhong, Lichen Yi, Yuemei Li

**Affiliations:** ^1^The First Clinical Medical College, Guangzhou University of Chinese Medicine, Guangzhou, China; ^2^Department of Rehabilitation, Guangzhou Eighth People’s Hospital, Guangzhou Medical University, Guangzhou, China

**Keywords:** acupuncture therapy, painful diabetic peripheral neuropathy, alternative and complementary medicine, meta-analysis, systematic review

## Abstract

**Background:**

Painful Diabetic Peripheral Neuropathy (PDPN) is a common complication of diabetes, it severely affects the quality of life of patients. Acupuncture has been shown to be effective in the treatment of PDPN. To evaluate the efficacy and safety of acupuncture for pain relief in patients diagnosed with diabetic peripheral neuropathy, we conducted a systematic review and meta-analysis.

**Method:**

We thoroughly searched specific databases, which included PUBMED, EMBASE, Web of Science, the Cochrane Library, the Chinese Biomedical Literature Database, the Chinese National Knowledge Infrastructure, China Science and Technology Journal Database and the Wanfang Data. All randomized controlled trials of acupuncture therapy for PDPN with pain change scales were included. Included studies were assessed for methodological quality according to the risk of bias from the Cochrane handbook. Meta-analyses were carried out to analyze the outcomes, subgroup analyses, sensitivity analyses, and funnel plot analyses were undertaken.

**Results:**

This systematic review evaluated a total of 25 trials of acupuncture therapy in combination with conventional treatment, involving a total of 1,561 patients with PDPN. According to the results, among 16 trials using VAS scores with a total of 1,552 patients, 2 acupoint injection trials (MD −2.38, 95% CI: −2.76 to −2.01, *p* < 0.00001), 12 acupuncture trials (MD −1. 31, 95% CI: −1.60 to −1.02, *p* < 0.00001) and 2 moxibustion trials showed that acupuncture therapy combined with conventional treatment improved pain better than conventional treatment (MD −2.50, 95% CI: −2.76 to −2.24, *p* < 0.00001). In the subgroup analysis of the acupuncture group, the results of the 5 trials in which the location of acupuncture was only in the limbs (MD −1.27, 95% CI: −1.54 to −1.01, *p* < 0.00001) and the 7 trials both in limbs and torso (MD −1.38, 95% CI: −1.81 to −0.95, *p* < 0.00001) also demonstrated that acupuncture was effective in pain improvement.

**Conclusion:**

This meta-analysis analyzed the possible efficacy of acupuncture in combination with conventional treatment for pain in diabetic peripheral neuropathy, particularly when acupoints are located in the limbs. However, there are limitations to this meta-analysis and future clinical studies are needed to confirm these findings.

**Systematic review registration:**

https://www.crd.york.ac.uk/prospero/display_record.php?ID=CRD42023449447, identifier (CRD42023449447).

## 1 Introduction

According to the International Diabetes Federation, approximately 425 million people worldwide suffer from diabetes. It is considered the most significant global epidemic of this century (1). The prevalence of diabetes in individuals between the ages of 20 and 79 was estimated to be 10.5% (536.6 million people) globally in 2021, with an expected rise to 12.2% (783.2 million people) by 2045 (2).

Individuals with diabetes mellitus may develop severe chronic complications. One of the most common complications is diabetic peripheral neuropathy. Diabetic neuropathy refers to the loss of sensory function starting distally in the lower extremities, accompanied by pain and significant morbidity. At least 50% of individuals with diabetes develop diabetic neuropathy over time (3), and over 30% of them may occur neuropathic pain (4). Unfortunately, pain may persist for years and can be challenging to treat because the underlying mechanisms are still unclear (5).

Painful diabetic peripheral neuropathy (PDPN) is characterized by numbness, burning, and tingling sensations around the hands and feet. The level of pain is generally classified as moderate to severe, it lasts for an extended period, and is exacerbated at night. This condition can significantly affect the quality of life, disrupt employment, impair sleep and lead to poor mental health, including high levels of depression and anxiety (6). In severe cases, PDPN may result in refractory ulcers that can lead to amputation, negatively affecting the quality of life of patients and ultimately increasing mortality, placing a significant burden on families and society (7, 8).

The current management approaches for diabetic neuropathy concentrate on controlling glycemia, implementing lifestyle changes such as diet and physical activity, and utilizing medication-based pain relief (9). However, the association between hyperglycemia and complications, including neuropathy, has been reported to be less convincing in patients, especially those with type 2 diabetes (10). This suggests that focusing on glycemic control alone may not be sufficient to mitigate diabetic neuropathy. Simultaneously, although there have been relentless efforts and continuous research, available medications for relieving diabetic neuropathy pain are only partially effective and have substantial side effects. This inadequacy is partly due to insufficient understanding of the complex underlying causes of PDPN (11). There is limited literature on pharmacologic and combination therapies for the prevention or reversal of diabetic peripheral neuropathy changes or for complete pain relief. Therapeutic management of PDPN has a number of unmet needs (12). Therefore, discovering a secure, trustworthy, and efficient non-pharmacological therapy might be a viable choice. Recently, acupuncture therapies have gained global recognition as a complementary alternative medicine, and are considered safe and well tolerated with few reported adverse effects. Acupuncture therapies are increasingly used as an integrative or complementary therapy for pain. Controlled trials have been published on acupuncture treatment for various pain syndromes, including acute and chronic low back pain, knee osteoarthritis, headache, myofascial pain, neck pain, and fibromyalgia (13). Acupuncture also has great advantages in treating neuropathic pain (14). Numerous animal experiments and clinical studies suggest that acupuncture primarily intervenes in neuropathic pain through the sensory, emotional, cognitive, and social dimensions (15).

Several randomized controlled trials (RCT) with small sample sizes have been conducted on acupuncture therapies for PDPN in recent years. However, to our knowledge, there are no systematic reviews or Meta-analyses offering a summary. Thus, additional research is required to fully understand this topic. This meta-analysis significantly advances our comprehension of the role of acupuncture therapies in PDPN. We hope that the study results will be intriguing to health care professionals, researchers and patients.

## 2 Materials and methods

### 2.1 Study registration

This protocol of systematic review and meta-analysis has been registered on Prospective Register of Systematic Reviews (PROSPERO) with number CRD42023449447. According to the guidance of the Preferred Reporting Items for Systematic Reviews and Meta-Analyses statement (PRISMA), we conducted and reported this systematic review.

### 2.2 Search strategy

Searches were conducted at the following databases by two independent authors (LZ and TW). If there is any disagreement, it will be resolved by discussion or decided by the third author.

The databases including the PUBMED, EMBASE, Web of Science, the Cochrane Library, the Chinese Biomedical Literature Database (CBM), the Chinese National Knowledge Infrastructure (CNKI), China Science and Technology Journal Database and the Wanfang Data. The search dates will be set from the inception to August 2023. The sample of the search strategy for PUBMED is presented in Supplementary Table 1.

### 2.3 Selection criteria

Studies were eligible for inclusion if they met the following criteria: (1) a randomized controlled trial design evaluating acupuncture therapies for diabetic peripheral neuropathy; (2) participants who experienced painful distress due to diabetic peripheral neuropathy; (3) acupuncture therapies, including traditional needling, electroacupuncture, auricular acupuncture, laser acupuncture, fire needling, acupoint injection, and moxibustion; (4) the control group consisting of conventional treatment, sham acupuncture, or blank control; (5) pain change scales including but not limited to Visual Analog Scale (VAS) score or Bodily Pain score on the quality of life [36-Item Short Form Health Survey (SF-36)]; and (6) language restrictions are English and Chinese.

The following types of studies were excluded: (1) non-randomized clinical studies, cluster randomized trials, and quasi-randomized trials conference abstracts; (2) case reports, protocols, reviews, and studies conducted on an animal or cellular level; (3) duplicated literature; (4) studies with insufficient data; (5) articles on herbal medicine, cupping, or any other external Chinese Medicine treatments not mentioned above; and (6) literature not published in either English or Chinese.

The primary outcomes will be the patient-reported pain intensity using VAS and Bodily Pain score on SF-36. The secondary outcomes including the Toronto Clinical Scoring System (TCSS) and clinical efficacy based on VAS scores, were categorized as effective and ineffective.

### 2.4 Data extraction

The data were independently collected by two researchers using Excel tables from every included study, and then reviewed by a third individual. The collected information included the first author’s name, year of publication, survey period, subjects, diagnostic criteria of diabetes, sample size, age, length of illness, intervention method, duration of intervention, frequency of treatment, course of treatment, outcome measures, and the selected acupoints of treatment. The outcomes of pain intensity were measured as continuous variables. The mean difference (MD) and standardized mean difference (SMD) before and after treatment was used to pool differences between experimental and control groups in each study.

### 2.5 Risk of bias assessment

The researchers independently assessed the studies by using the Cochrane Risk of Bias2 (RoB2) tool. The related criteria cover: random sequence generation, allocation concealment, blinding of participants and personnel, blinding of outcome assessment, incomplete outcome data, selective reporting, and other bias. Each evaluation was classified as low, unclear, or high risk of bias. If a conflict arises, a third researcher assists in reaching a consensus.

### 2.6 Statistical analysis

All analyses were conducted using RevMan 5.3. If the extracted data was not sufficient to perform a meta-analysis, a qualitative analysis was carried out instead. The study results were standardized into a unified scale by using the standardized mean difference (SMD) and the 95% confidence interval (CI) to analyze the outcome data. SMD with 95% CI was calculated with heterogeneity tested by the I^2^ test. Data was combined by a fixed effect model when I^2^ < 50%. Otherwise, a random effect model was applied. There was a significant difference if the value of p was <0.05 between the two groups. Subgroup analysis or sensitivity analysis could help find out the sources of heterogeneity. Moreover, a descriptive analysis was carried out when the causes of heterogeneity could not be determined. A funnel plot was used to evaluate the publication bias.

### 2.7 Quality of evidence

The overall quality of outcomes was graded using the GRADEpro GDT classification of the following domains: study design, risk of bias, inconsistency, indirectness, imprecision, and other considerations.

## 3 Results

### 3.1 Study selection

**Figure 1:**
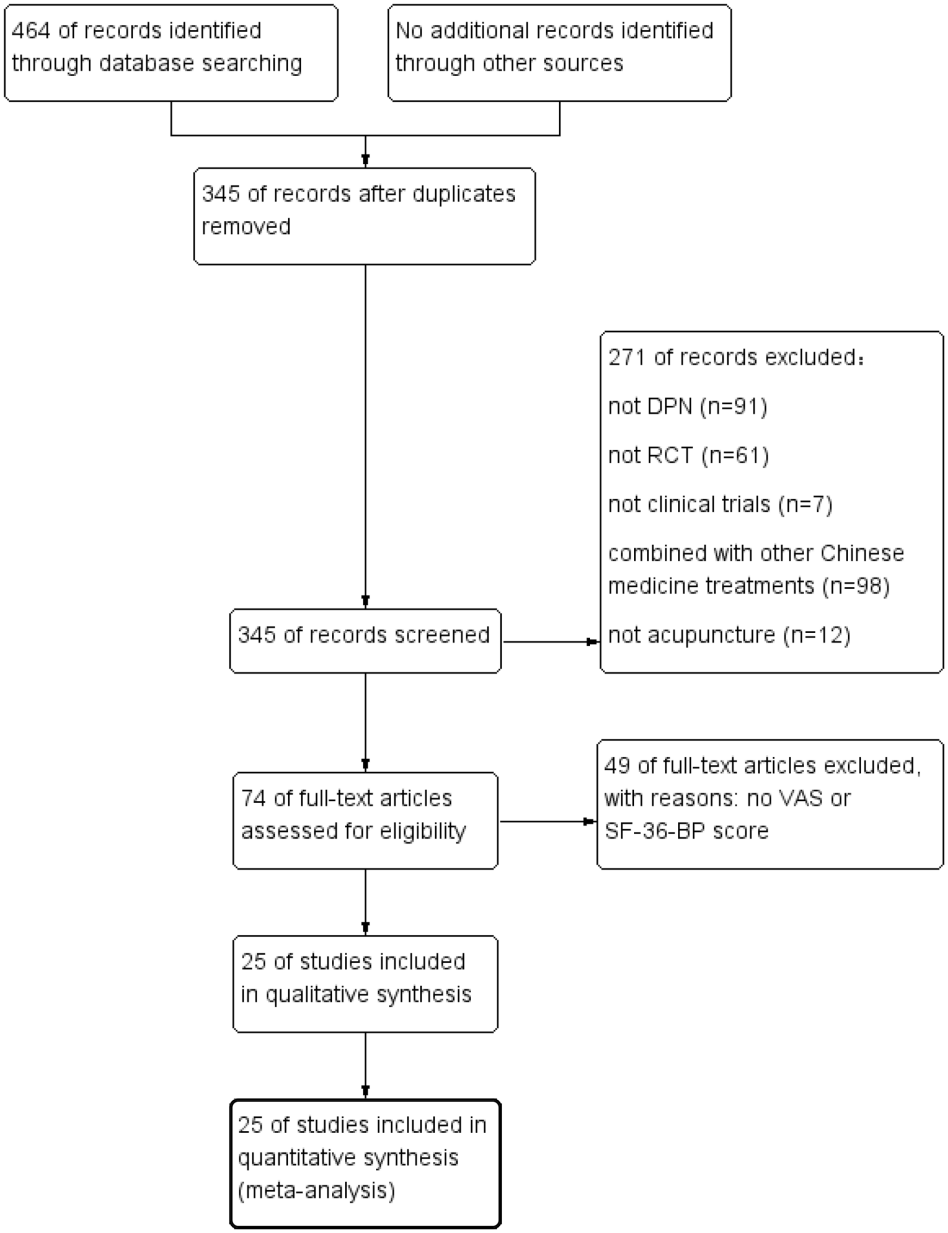
Study flow diagram.

464 studies were obtained from the database search. After the removal of the 119 duplicate articles, 271 articles were excluded, which were considered to be irrelevant to our research. The full text of the remaining 74 articles was screened. Excluding 49 reports without VAS or SF-36 Bodily Pain scores, 25 studies in Chinese and English finally met the inclusion criteria. This study included a total of 1,561 patients with diabetic peripheral neuropathy. A meta-analysis was conducted on these articles. Figure 1 displays the process of trial selection.

### 3.2 Description of the included studies

All of the included studies were single-center RCTs, with one conducted in the United States (16), and the remaining 24 in China (17–40). All participants in the studies were diagnosed with diabetic peripheral neuropathy. The acupuncture therapies included the following interventions, 2 for acupoint injection (17, 28), 12 for manipulative needling (16, 19, 25–27, 29–32, 34, 37, 40), 2 for warm needling combined with manipulative needling (22, 35), 3 for moxibustion (20, 33, 38), 2 for electroacupuncture (36, 39), 1 for acupoint injection combined with warm needling (18), and 3 for manipulative needling combined with warm needling (21, 23, 24). All the studies included had either VAS or SF-36 Bodily Pain scores as primary outcome indicators, with treatment durations ranging from 6 days to 3 months and treatment time ranging from 20 to 45 min. Moreover, all studies reported positive effects. Detailed information can be found in Supplementary Table 2.

### 3.3 Risk of bias within trials

Out of the included articles, 14 studies were classified as low risk for randomization sequence generation, with 1 trial (16) using a computer-generated randomization list, 1 trial (37) using a simple lottery randomization method, 12 trials (17–19, 21, 23, 26, 28, 31, 34, 35, 38, 39) using a table of random numbers. 8 trials (20, 24, 25, 27, 29, 30, 32, 36) lacking detailed information, resulting in an unclear risk of randomization bias. 3 trials (22, 33, 40) with errors in the randomization methods were classified as high risk.

**Figure 2:**
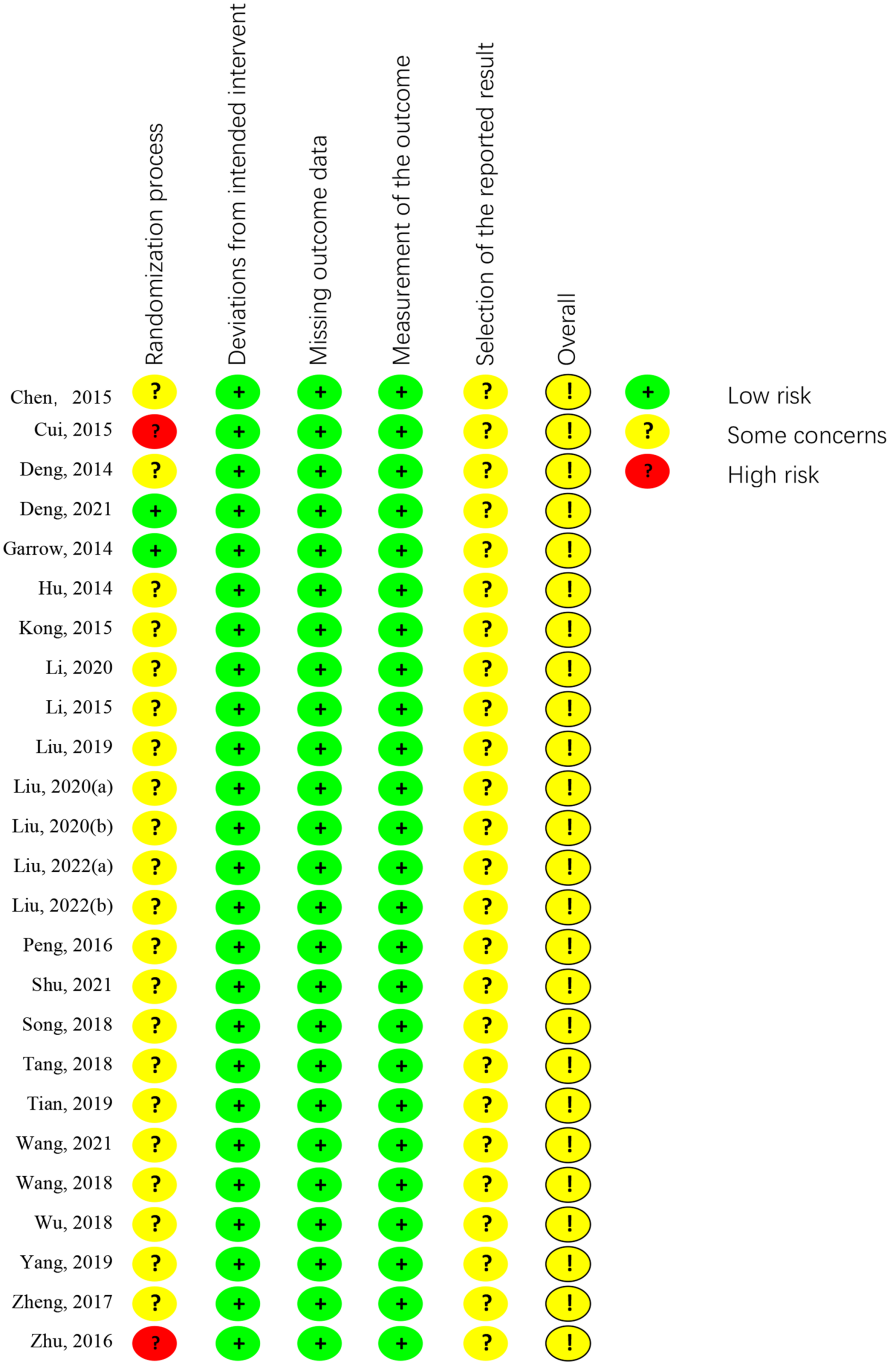
Risk of bias summary.

**Figure 3:**
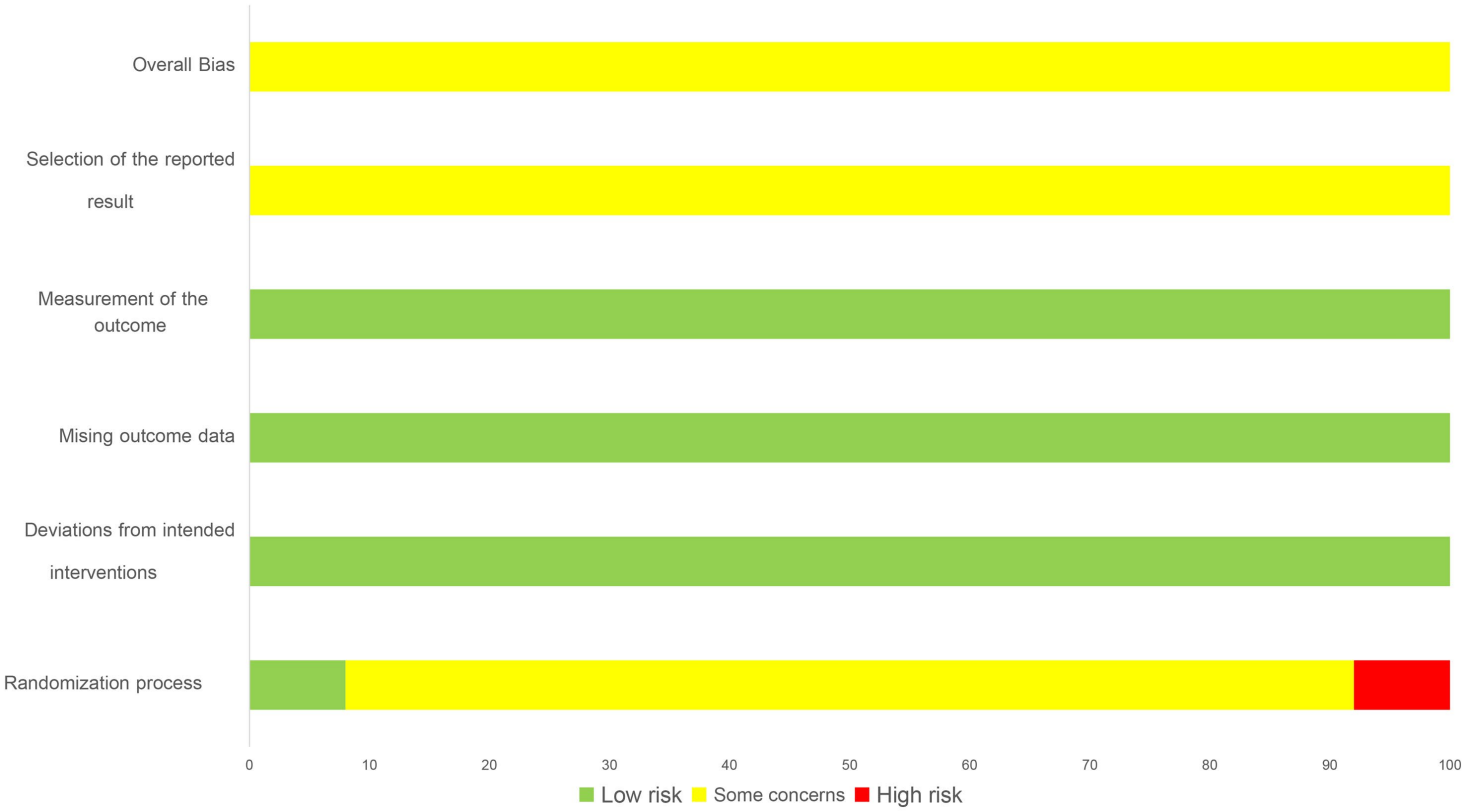
Risk of bias graph.

Two trials (16, 19) was assessed as having a low risk of allocation concealment bias since a computer-generated list of random numbers was placed in a sealed opaque envelope. The remaining 23 trials (17, 18, 20–40) were not described in sufficient detail and were considered to have an unclear risk of allocation concealment bias. None of the trials employed double-blind procedures, as the acupuncturists were not blinded. In one trial (16), the assessor-blind method was described in the results, while the other trials did not mention the blinding of outcome assessment. Out of the 25 trials evaluated, 24 reported the expected study outcome and presented complete outcome data, whereas 1 trial (32) did not report complete data and was evaluated as having an unclear risk. However, as this was not the primary outcome, it can still be systematically evaluated. All trials were graded as having an unclear risk among other sources of bias. The summary information is shown in Figures 2, 3.

### 3.4 Effects of interventions

#### 3.4.1 Primary outcome (VAS and SF-36 bodily pain)

**Figure 4:**
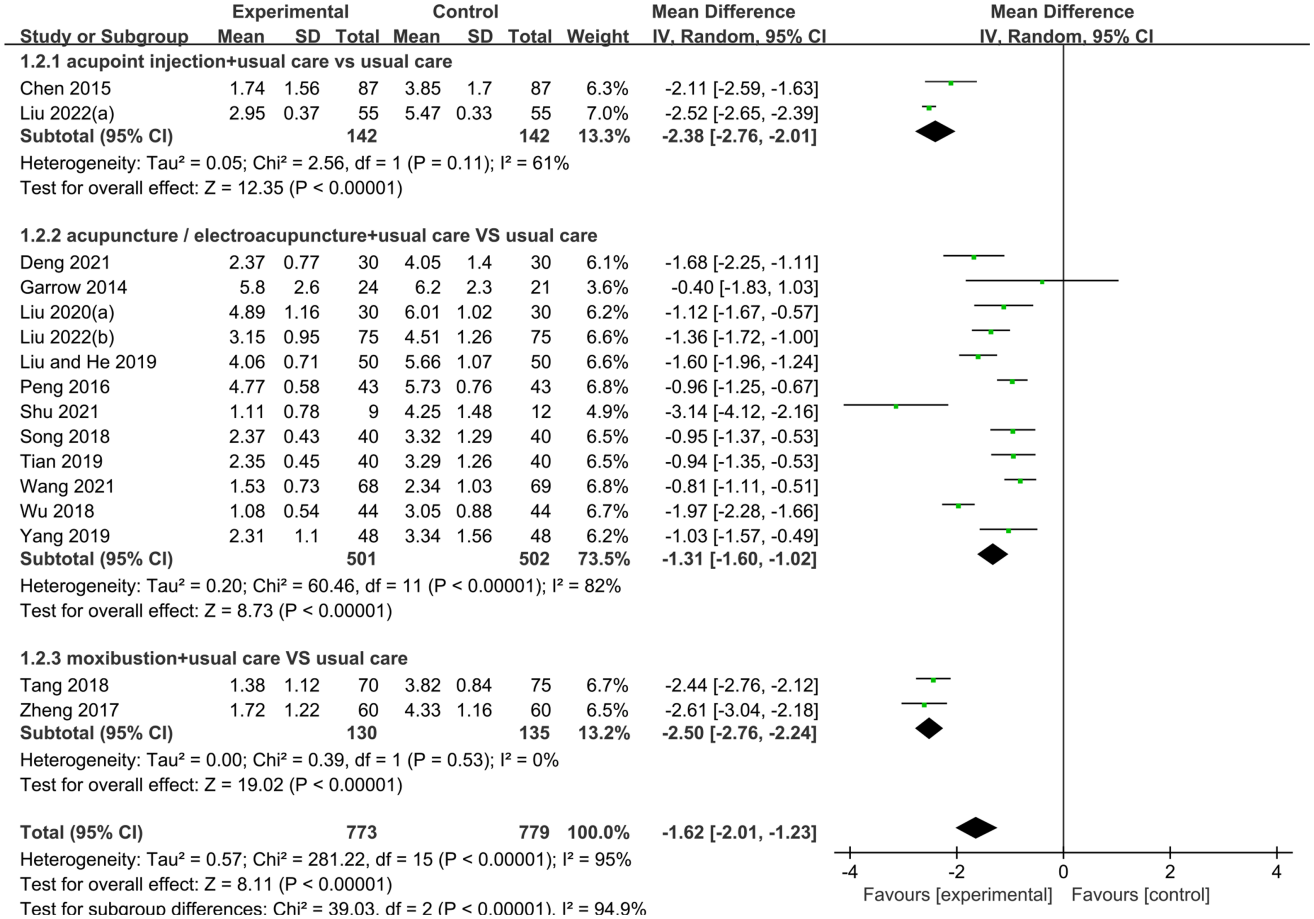
Forest plot and meta-analysis of VAS Score.

**Figure 5:**
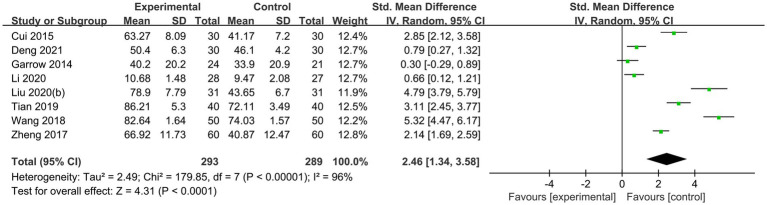
Forest plot and meta-analysis of SF-36 Bodily Pain score.

20 randomized controlled trials (16, 17, 19, 20, 22, 23, 26–39) explored the improvement of pain in diabetic peripheral neuropathy with acupuncture therapies, of which 16 trials (16, 17, 19, 23, 26–34, 37–39) were assessed with VAS scores, including 2 acupoint injection trials (17, 28), 12 needling trials (16, 19, 26, 27, 29–32, 34, 36, 37, 39) and 2 moxibustion trials (33, 38). A random effects model (*p* < 0.001, I^2^ = 95%) was used for the results, and there was a significant effect of pain improvement in the acupuncture therapy group (MD −1.62, 95% CI: −2.01 to −1.23, *p* < 0.00001; Figure 4). 8 trials used the SF-36 Bodily Pain score, including 2 trials of needling combined with moxibustion (22, 35), 2 trials of moxibustion (20, 38), 3 trials of needling (16, 19, 34) and 1 trial of warm needling combined with needling (23). The results were analyzed using a random effects model (*p* < 0.0001, I^2^ = 96%) and showed that acupuncture therapies significantly improved pain (SMD 2.44, 95% CI: 1.33 to 3.56, *p* < 0.001; Figure 5).

#### 3.4.2 Secondary outcome (effective rate and TCSS)

**Figure 6:**
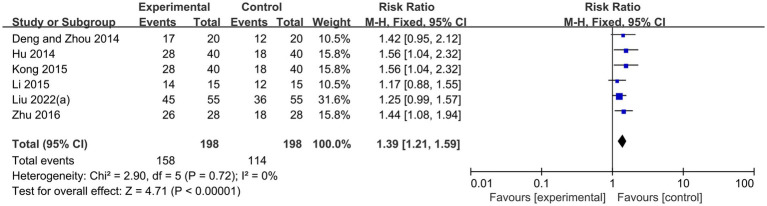
Forest plot and meta-analysis of effective rate.

**Figure 7:**
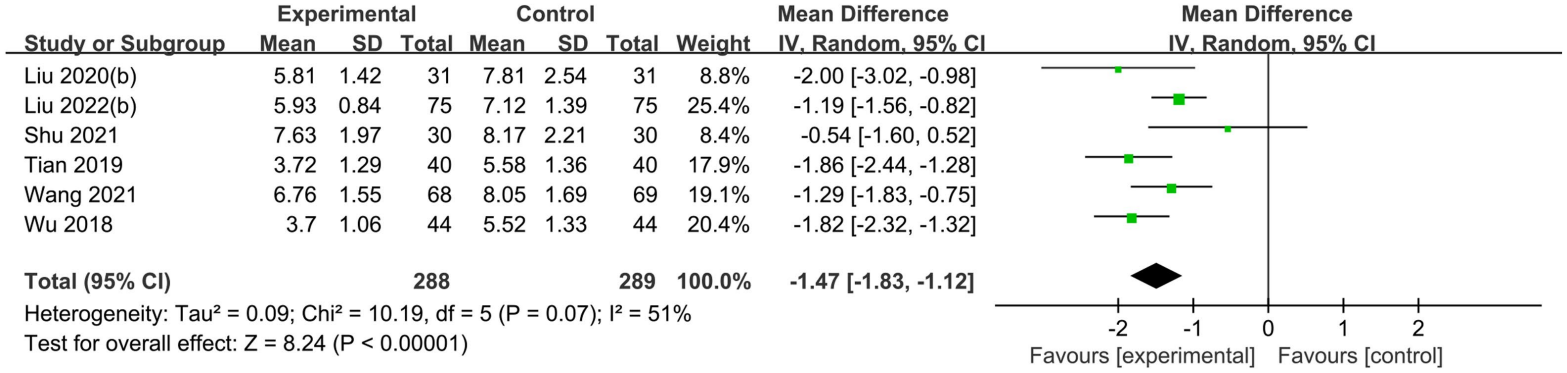
Forest plot and meta-analysis of TCSS Scores.

Six randomized controlled trials (18, 21, 24, 25, 28, 40), comprising 396 patients, statistically examined clinical efficacy based on VAS scores, investigated the use of acupuncture therapies to alleviate pain caused by diabetic peripheral neuropathy. Using a fixed-effects model, the results showed that acupuncture therapy combined with conventional therapy has significantly higher effective rate than conventional therapy alone (RR 1.39, 95%CI: 1.21 to 1.59, *P* < 0.00001, I^2^ = 0%; Figure 6). Another six RCTs (23, 27, 29, 31, 34, 36) were conducted involving 577 patients, using a random effects model. These trials showed that when acupuncture therapy was combined with conventional therapy for diabetic peripheral neuropathy, the TCSS scores were higher compared to conventional therapy alone (MD −1.47, 95%CI: −1.83 to −1.12, *P* < 0.00001, I^2^ = 51%; Figure 7).

#### 3.4.3 Subgroup analysis

Subgroup analyses were used to test whether different acupuncture methods affected the improvement of PDPN. 2 acupoint injection trials (17, 28) with 284 patients, using a random-effects model, showed that acupoint injections in combination with conventional therapies were more effective than conventional therapies in relieving pain (MD −2.38, 95% CI: −2.76 to −2.01, *p* < 0.00001, I^2^ = 61%). 12 needling trials (16, 19, 26, 27, 29–32, 34, 36, 37, 39) with a total of 1,003 patients, using a random effects model, showed that needling combined with conventional therapy was more effective than conventional therapy in improving pain (MD −1.31, 95% CI: −1.60 to −1.02, *p* < 0.00001, I^2^ = 82%). 2 moxibustion trials (33, 38) with a total of 265 patients, using a random effects model, showed that moxibustion combined with conventional therapy was more effective than conventional therapy in improving pain (MD −2.50, 95% CI: −2.76 to −2.24, *p* < 0.00001, I^2^ = 0%; Figure 4).

**Figure 8:**
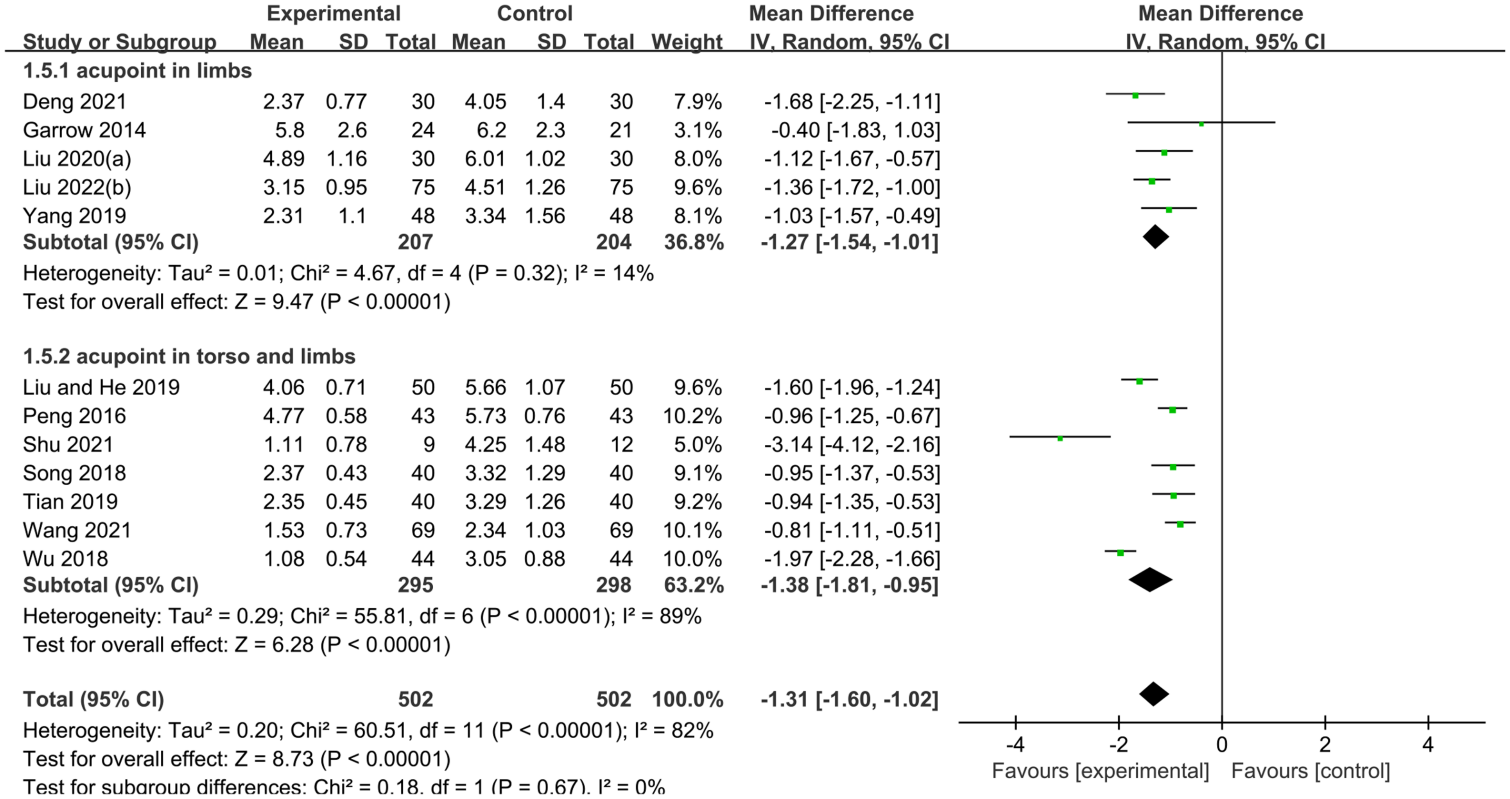
Forest plot and Sub group analysis of VAS Score.

To verify the effectiveness of needling combined with conventional therapy in improving pain in diabetic peripheral neuropathy, a subgroup analysis was conducted to compare the outcomes with different needling locations. Among 5 studies (16, 19, 26, 27, 30) with a total of 411 patients and needling sites on the limbs, a random-effects model showed that needling combined with conventional therapy was more effective than conventional therapy in improving pain (MD −1.27, 95% CI: −1.54 to −1.01, *p* < 0.00001, I^2^ = 14%). Among the 7 studies (29, 31, 32, 34, 36, 37, 39) with a total of 593 patients, with needling sites on the torso and limbs, using a random effects model, the results showed that needling combined with conventional therapies was more effective than conventional therapies in improving pain (MD −1.38, 95% CI: −1.81 to −0.95, *p* < 0.00001, I^2^ = 89%; Figure 8).

#### 3.4.4 Adverse events

Safety conditions was reported in all 25 RCTs (16–40), and adverse effects were not mentioned in 15 of them (21, 22, 24–32, 34, 35, 38, 40). Out of the 10 studies which mentioned adverse effects, only two trials (16, 18) reported a negative association with acupuncture therapy while the other eight trials (17, 19, 20, 23, 33, 36, 37, 39) reported none. The adverse effects observed were mainly pain, discomfort, and swelling, all of which were mild and showed signs of reversibility. Details are shown in Supplementary Table 2.

#### 3.4.5 Sensitivity analysis and publication bias

**Figure 9:**
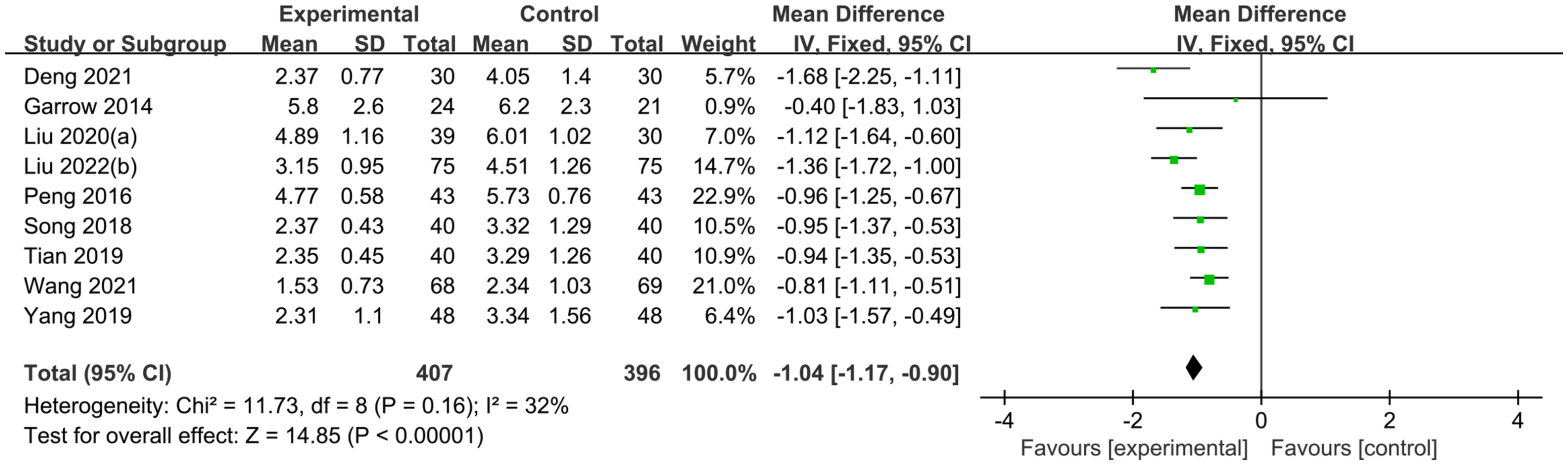
Sensitivity analysis. Forest plot and meta-analysis of VAS Score after removing the studies of Shu et al. (31), Liu and He (39), and Wu et al. (29).

**Figure 10:**
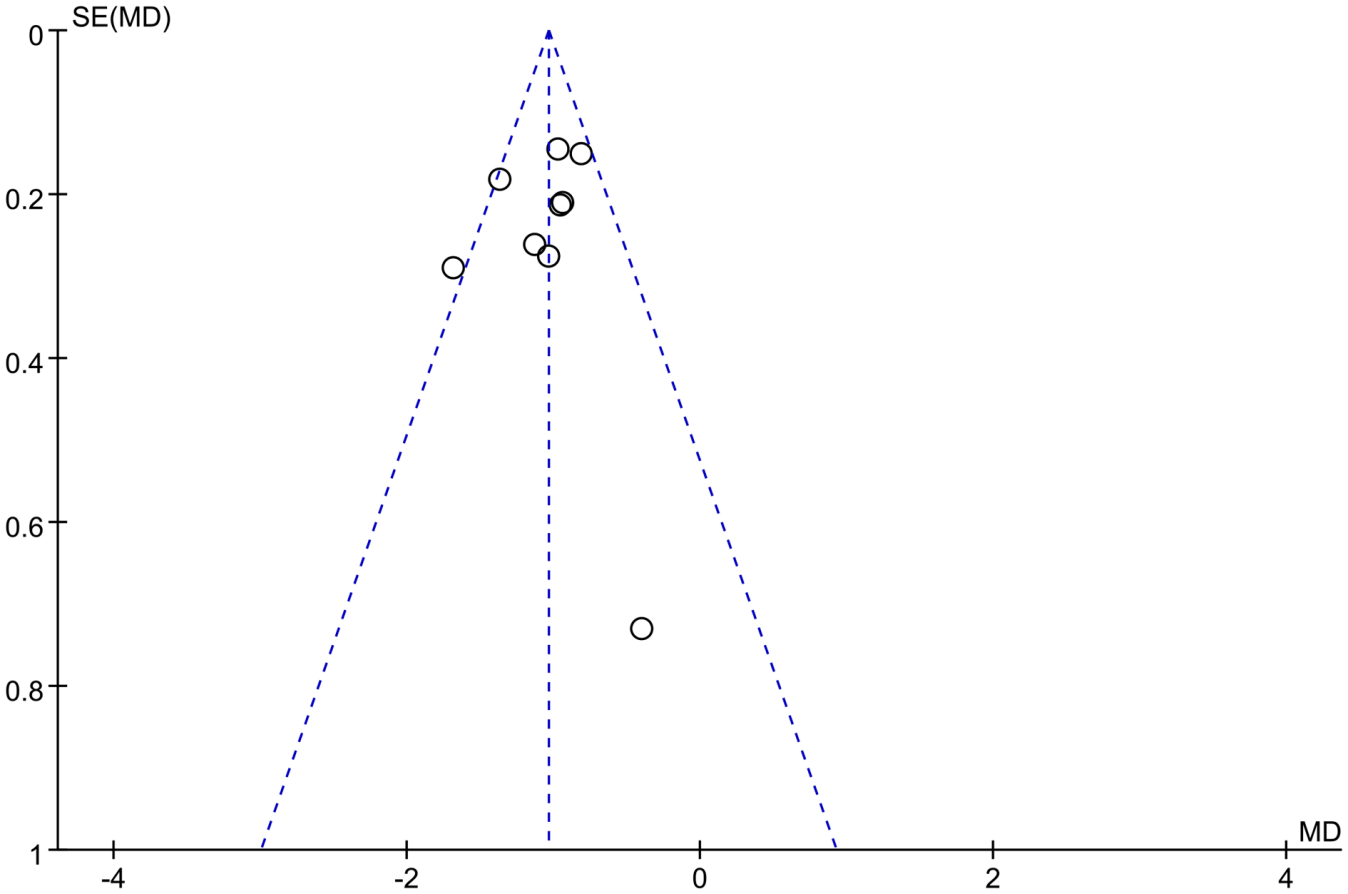
Funnel plots of VAS scores.

The sensitivity analyses illustrated that the studies conducted by Shu et al. (31), Liu and He (39), and Wu et al. (29) may be the primary source of heterogeneity in the acupuncture group of VAS scores, as I^2^ decreased to 32% after their removal (Figure 9). The funnel plot of pain improvement was asymmetric, suggesting possible publication bias (Figure 10).

#### 3.4.6 Quality assessment

We used GRADE (Grading of Recommendations Assessment Development and Evaluation) tool to assess the available evidence, and the quality of evidence on acupuncture therapies for PDPN was “low.” Details are given in Supplementary Table 3.

## 4 Discussion

This study conducted a meta-analysis of 25 randomized controlled trials involving 1,561 subjects to assess the improvement of pain in DPN with acupuncture therapies. Qualitative analysis of the systematic reviews showed the effectiveness of acupuncture therapies in reducing pain in patients with DPN, and also the relief of neurosensory symptoms in the limbs. Subgroup analyses according to different acupuncture methods are usually performed to reduce the resulting risk of bias toward specific effects. The results of subgroup analyses demonstrated that different acupuncture methods in combination with conventional treatment were all more effective in improving pain than conventional treatment alone. Further subgroup analyses based on the needling location could lead to the same conclusion.

Diabetic peripheral neuropathy, a frequently occurring long-term complication of diabetes, affects 60% to 90% of individuals with diabetes (41), causing abnormal neurosensory perception and loss of function as its typical symptoms. Research (42) has indicated that up to 50% of individuals with diabetic peripheral neuropathy may experience diabetic peripheral neuropathic pain. Individuals with diabetic peripheral neuropathic pain are at risk of losing balance and coordination, being unable to perceive injuries, and falling (43). Research has demonstrated (44, 45) that chronic pain significantly impacts the physical and mental health of individuals, leading to personal, familial, and social issues such as depression, suicide, unemployment, and isolation. These issues directly affect the quality of life of affected individuals. Pain, including neuropathic pain, nociceptive allergy, and nociceptive hypersensitivity, as one of the pathological subjective sensations of this disease, varies in individual understanding and perception of pain. Therefore, the VAS score and SF-36 score, as commonly used pain assessment scales in the international community, can be used to assess pain from a single dimension or multiple dimensions. The self-rating scale assessment method is a simple, convenient, economical and tested assessment tool and is considered the gold standard for pain assessment (46). Additionally, the TCSS score can serve as a standardized method of assessing the severity of neurological signs and symptoms (47). Therefore, this study evaluated the effectiveness of acupuncture therapies on PDPN by VAS, SF-36 Bodily Pain and TCSS score.

The potential mechanisms of acupuncture for treating sensory nerve dysfunction in patients with PDPN are the following. Hyperglycemia-induced oxidative stress can potentially play a role in the development of pain, either directly or indirectly (5). Hyperglycemia leads to the production of reactive substances such as ROS (including NOX2-produced superoxide and NOX4) and glycosylation end products (AGE). These substances can directly increase pain signaling (48). Studies conducted by Sun et al. (49) and He et al. (50) have shown that electroacupuncture and acupoint injection can augment superoxide dismutase (SOD) levels in both the serum and sciatic nerves of rats. This finding suggests that acupuncture can alleviate pain signaling by increasing SOD levels and breaking down ROS and AGE end products. Su et al. (51) discovered that warm needing effectively inhibits the expression of sciatic nerve messenger RNAs for glycosylation end-products (AGEs mRNA) and glycosylation end-product receptors (RAGE mRNA), providing genetic evidence that warm acupuncture suppresses non-enzymatic protein glycosylation within the sciatic nerve. In addition, hyperglycemia leads to elevated levels of glutamate, the primary excitatory neurotransmitter in the central nervous system. Glutamate is released by both nociceptors and spinal projection neurons to transmit pain signals (52). Li et al. (53) found that electroacupuncture reduced pain by reducing spinal glutamate levels. Furthermore, electroacupuncture was utilized by Ma et al. (54) to treat PDPN rats and resulted in inhibition of the activation of the p38MAPK signaling pathway in the spinal cord, leading to reduced pain in the rats with PDPN. Also, relevant studies have shown that the release of pro-inflammatory factors and inflammatory response is a cause of pain (55, 56). Acupuncture can make serum TNF-α, IFN-γ show a downward trend, reduce the infiltration of peripheral nerve tissue, reduce the sensitivity of mechanical pain and thermal pain, and alleviate the symptoms of pain (31). Acupuncture can reduce the level of serum high sensitivity CRP (hs-CRP), accelerate the absorption of products of local injury, necrosis and disintegration, and reduce the level of pain (57). In a study of electroacupuncture, it was found that by inhibiting the activity of lipoxygenase LOX, the neuroinflammatory response was reduced, which in turn led to a decrease in the mechanical pain sensitivity of DPN rats (58). In addition, observation after needling DPN model rats showed that needling could normalize nerve fiber arrangement, inhibit myelin demyelination and reduce the release of pain-causing neurotransmitters (59). Dong et al. (60) established an animal model of diabetic peripheral neuropathy wherein the rats in the needling group exhibited elevated thermal pain threshold. Zhou et al. (61) found that electroacupuncture can also provide analgesia mediated by inhibiting PKC-dependent membrane P2X3 upregulation in DRG. In addition, late development of PDPN leads to demyelination of peripheral nerves, resulting in nervous system abnormalities and deficits, with the degree of demyelination closely linked to pain hypersensitivity (62). A meta-analysis of acupuncture for neuropathic pain suggested that the adverse effects of acupuncture for neuropathic pain were mild and reversible (63). The mechanism of acupuncture’s action on pain in patients with DPN is not entirely clear. Therefore, further research is warranted to concentrate on how acupuncture improves PDPN.

**Figure 11:**
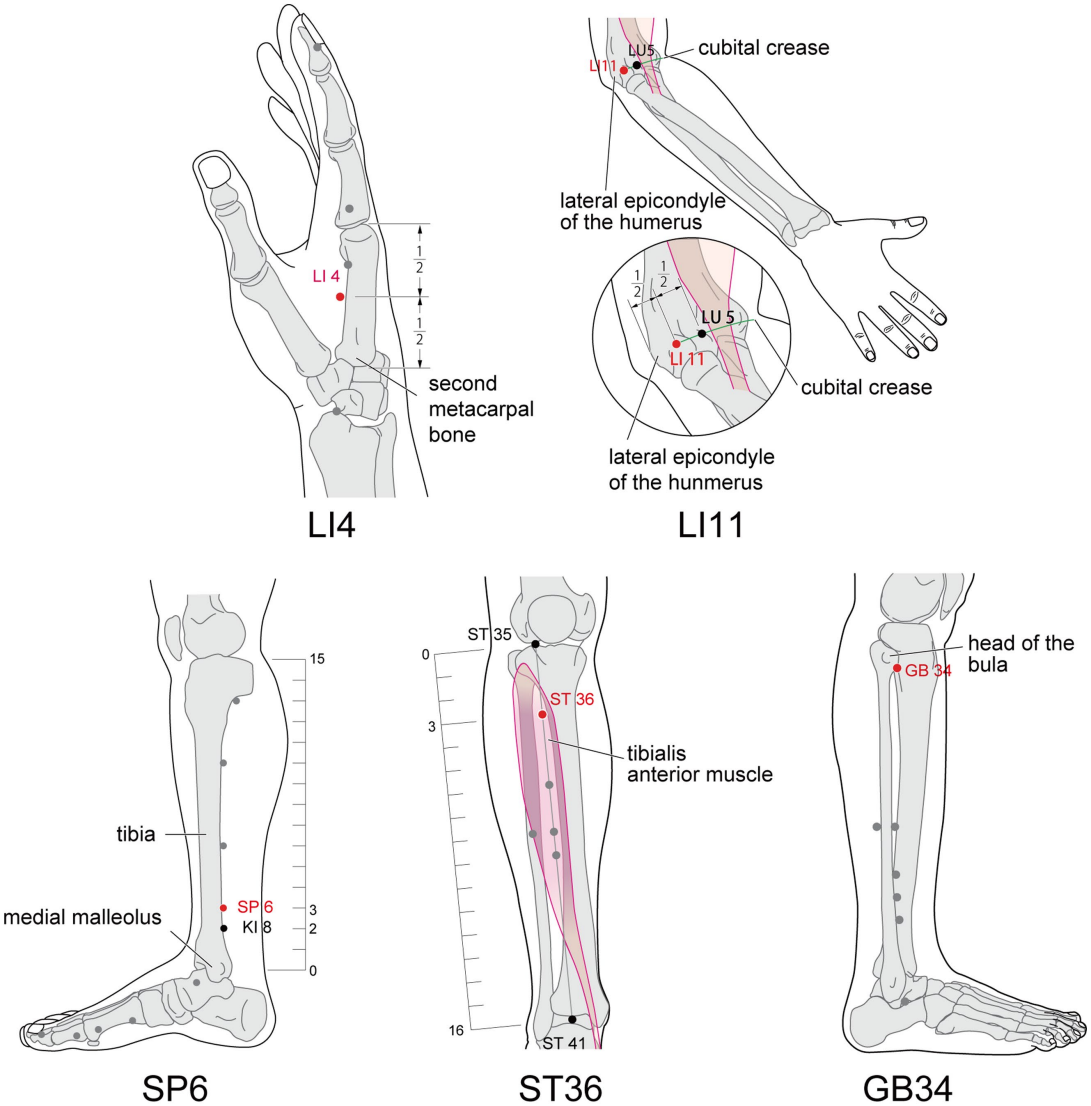
The acupoints location of ST 36, GB34, SP6, LI11, and LI4.

Studies (64) has shown that the most commonly used analgesic acupoints on the PDPN model are zusanli (ST36), kunlun (BL60), quchi (LI11), hegu (LI4), sanyinjiao (SP6), and yanglingquan (GB34), all of which are located in the limbs. Similarly, in the studies we included, the five acupoints that appeared most frequently were ST36, SP6, LI4, LI11, GB34 (Figure 11). The affected area of Diabetic Peripheral Neuropathy (DPN) receives innervation primarily from the cervical, lower thoracic, and lumbosacral nerves. All of the above acupoints all exhibit central location within the ganglionic innervation zones of C5~T1, T11~T12, and L2~S3. Therefore, the ganglionic connection between the aforementioned acupoints and the affected area of DPN provides the physiological foundation for treating the disease (65). Based on the above views, we performed subgroup analyses according to the location of needling point distribution, and the results showed that the efficacy of pain improvement in the limbs needling group was significant and the heterogeneity was low. However, when the acupoints distributing the extremities were combined with those of the torso, the heterogeneity remained high, and we considered that this might be due to the fact that the acupoints of the torso were treated as additional allocation points, and that the relative diversity of acupoint selection between studies was the main source of heterogeneity. In conclusion, acupuncture therapies may be able to be used as complementary alternative therapies for the treatment of PDPN.

### 4.1 Study strengths and limitations

The study has various strengths. First, previous systematic evaluations of acupuncture for diabetic peripheral neuropathy have mainly assessed its efficacy in terms of nerve conduction, with only a few studies conducted on pain improvement. Second, our review, which focused on acupuncture therapy alone (or in combination with modern medicine medications), excluded studies of other Chinese medicine mixed therapies, which are more likely to reflect the efficacy of acupuncture *per se*. Third, we included a higher number of RCTs with larger sample sizes and more acupuncture operations; Fourth, we performed subgroup analyses according to the distribution of the location of acupoints to provide a basis for guiding clinical treatment. Fifth, we also performed sensitivity analyses and evaluated publication bias using funnel plots. Sixth, all the trials we included used the same pain scoring tool, which reduces bias due to different scoring criteria. Thus, our study provides reference evidence supporting the use of acupuncture therapies in clinical practice.

However, there are some limitations to this study. The majority of the trials included in this study were conducted in China, indicating potential racial bias. Moreover, due to the specificity of acupuncture treatment, it is difficult to achieve blinding of participants and operators during the study. Furthermore, the large heterogeneity observed among the included articles may be attributable to the following reasons: (i) the sample size of the included articles was small; (ii) there were differences in the professional level of acupuncturists, the needling technique, the selection of acupuncture points, the composition of moxibustion drugs and the frequency of electroacupuncture, and some studies (64) suggested that although different frequencies of electroacupuncture were effective in the rat model of DPN, the low-frequency efficacy was better; (iii) Patients included in the trials had different initial pain levels, resulting in possible clinical heterogeneity between studies; (iv) The duration of acupuncture treatment and the intensity of intervention were not exactly the same across all studies. Most of the studies had a short intervention time, and their results only reflect the effect of short-term acupuncture treatment for PDPN. Therefore, the need for more large-sample multicenter long-term RCTs is warranted. To summarize, this paper demonstrates the therapeutic effectiveness of acupuncture therapies for PDPN. Yet, the specific most effective needling technique, rational point selection and moxibustion drug composition still need further research. These deficiencies need to be addressed in future studies. We also hope to design better RCT studies in the future to further validate the conclusions drawn from this research.

### 4.2 Implication for further research

Our findings suggest a need for further high-quality studies on acupuncture for patients with PDPN to increase the sample size and minimize bias. Observing the long-term effects of acupuncture treatment requires longer follow-up trials. Future studies should follow the Comprehensive Criteria for Reporting Trials (CONSORT) statement and a strict checklist (66, 67). To achieve a double-blind, standardized trial design, timely data storage and a well-coordinated team are necessary to successfully implement the sham intervention.

## 5 Conclusion

After assessing different pain rating scales, it is concluded that acupuncture therapies possibly improve pain caused by diabetic peripheral neuropathy. The curative effect may be more pronounced at certain acupoints located on the limbs. Due to the lack of clarity on the mechanism of acupuncture for treating PDPN, and the “low” GRADE for pain improvement in our study, this may suggest that the level of recommendation for this treatment should be “low” for clinical practice. We urge caution in interpreting our results due to the low methodological quality of the studies included in the selected publications.

## Data availability statement

The original contributions presented in the study are included in the article/Supplementary material, further inquiries can be directed to the corresponding author.

## Author contributions

LZ: Writing – original draft, Writing – review & editing. TW: Writing – original draft, Writing – review & editing. ZZ: Writing – original draft. LY: Writing – original draft. YL: Writing – review & editing.
