# Supplementary material for: Acupuncture for painful diabetic peripheral neuropathy: a systematic review and meta-analysis
Source: Front Neurol. 2023 Nov 16;14:1281485. doi: 10.3389/fneur.2023.1281485 (PMC10690617; doi:10.3389/fneur.2023.1281485)
Supplement: Supplementary file 1 [file Table_1.DOC]

| 1# | Acupuncture Therapy"[Mesh] / Acupuncture"[Mesh] OR (((((((((((((((((((((Pharmacopuncture[Title/Abstract]) OR (Acupuncture Treatment[Title/Abstract])) OR (Acupuncture Treatments[Title/Abstract])) OR (Treatment, Acupuncture[Title/Abstract])) OR (Therapy, Acupuncture[Title/Abstract])) OR (Pharmacoacupuncture Treatment[Title/Abstract])) OR (Treatment, Pharmacoacupuncture[Title/Abstract])) OR (Pharmacoacupuncture Therapy[Title/Abstract])) OR (Therapy, Pharmacoacupuncture[Title/Abstract])) OR (Acupotomy[Title/Abstract])) OR (Acupotomies[Title/Abstract])) OR (electroacupuncture[Title/Abstract])) OR (warm needling[Title/Abstract])) OR (moxibustion[Title/Abstract])) OR (cupping[Title/Abstract])) OR (auricular needle[Title/Abstract])) OR (eye acupuncture[Title/Abstract])) OR (scalp acupuncture[Title/Abstract])) OR (body acupuncture[Title/Abstract])) OR (acupoint[Title/Abstract])) OR (medicinal vesiculation[Title/Abstract])) OR (crude herb moxibustion[Title/Abstract]) |
| --- | --- |
| 2# | Diabetic Neuropathies"[Mesh] OR ((((((((((((((((((((((Diabetic Neuropathy[Title/Abstract]) OR (Neuropathies, Diabetic[Title/Abstract])) OR (Neuropathy, Diabetic[Title/Abstract])) OR (Diabetic Autonomic Neuropathy[Title/Abstract])) OR (Autonomic Neuropathies, Diabetic[Title/Abstract])) OR (Autonomic Neuropathy, Diabetic[Title/Abstract])) OR (Diabetic Autonomic Neuropathies[Title/Abstract])) OR (Neuropathies, Diabetic Autonomic[Title/Abstract])) OR (Neuropathy, Diabetic Autonomic[Title/Abstract])) OR (Diabetic Neuralgia[Title/Abstract])) OR (Diabetic Neuralgias[Title/Abstract])) OR (Neuralgias, Diabetic[Title/Abstract])) OR (Diabetic Neuropathy, Painful[Title/Abstract])) OR (Diabetic Neuropathies, Painful[Title/Abstract])) OR (Neuropathies, Painful Diabetic[Title/Abstract])) OR (Neuropathy, Painful Diabetic[Title/Abstract])) OR (Painful Diabetic Neuropathies[Title/Abstract])) OR (Painful Diabetic Neuropathy[Title/Abstract])) OR (Neuralgia, Diabetic[Title/Abstract])) ) OR (Symmetric Diabetic Proximal Motor Neuropathy[Title/Abstract])) OR (Diabetic Asymmetric Polyneuropathy[Title/Abstract])) OR (Polyneuropathies, Diabetic[Title/Abstract]) |
|  | 1.Development and validation of the Diabetic Peripheral Neuropathic Pain Impact (DPNPI)  2.visual analogue scale  3. 36-item Short-Form  4. toronto clinical scoring system |

**Table 1 Search Strategy**

**Pubmed**

**Embase**

| 1# | 'acupuncture'/exp OR 'Pharmacopuncture':ab,ti OR 'Acupuncture Treatment':ab,ti OR 'Acupuncture Treatments':ab,ti OR 'Treatment, Acupuncture':ab,ti OR 'Therapy, Acupuncture':ab,ti OR 'Pharmacoacupuncture Treatment':ab,ti OR 'Treatment, Pharmacoacupuncture':ab,ti OR 'Pharmacoacupuncture Therapy':ab,ti OR 'Therapy, Pharmacoacupuncture':ab,ti OR ‘Acupotomy’:ab,ti OR 'electroacupuncture':ab,ti OR 'warm needling':ab,ti OR 'moxibustion':ab,ti OR 'cupping':ab,ti OR 'auricular needle':ab,ti OR 'eye acupuncture':ab,ti OR 'scalp acupuncture':ab,ti OR 'body acupuncture':ab,ti OR 'acupoint':ab,ti OR 'medicinal vesiculation':ab,ti OR 'crude herb moxibustion':ab,ti |
| --- | --- |
| 2# | 'diabetic neuropathy'/exp OR 'diabetes neuropathy':ab,ti OR 'diabetic mononeuritis':ab,ti OR 'diabetic mononeuropathy':ab,ti OR 'diabetic neuritis':ab,ti OR 'diabetic neuropathies':ab,ti OR 'diabetic peripheral neuropathy':ab,ti OR 'diabetic polyneuritis':ab,ti OR 'diabetic polyneuropathy':ab,ti OR 'diabetic neuropathy':ab,ti |
|  |  |

**Cochrane**

| 1# | MeSH descriptor: [Acupuncture] explode all trees |
| --- | --- |
| 2# | Pharmacopuncture OR Acupuncture Treatment OR Acupuncture Treatments OR Acupotomy OR electroacupuncture OR warm needling OR moxibustion OR cupping OR auricular needle OR eye acupuncture OR scalp acupuncture OR body acupuncture OR acupoint OR medicinal vesiculation OR crude herb moxibustion |
| 3# | 1# OR 2# |
| 4# | MeSH descriptor: [Diabetic Neuropathies] explode all trees |
| 5# | Diabetic Neuropathy OR Neuropathies, Diabetic OR Neuropathy, Diabetic OR Diabetic Autonomic Neuropathy OR Autonomic Neuropathies, Diabetic OR Autonomic Neuropathy, Diabetic OR Diabetic Autonomic Neuropathies OR Neuropathies, Diabetic Autonomic OR Neuropathy, Diabetic Autonomic OR Diabetic Neuralgia OR Diabetic Neuralgias OR Neuralgias, Diabetic OR Diabetic Neuropathy, Painful OR Diabetic Neuropathies, Painful OR Neuropathies, Painful Diabetic OR Neuropathy, Painful Diabetic OR Painful Diabetic Neuropathies OR Painful Diabetic Neuropathy OR Neuralgia, Diabetic OR Symmetric Diabetic Proximal Motor Neuropathy OR Diabetic Asymmetric Polyneuropathy OR Polyneuropathies, Diabetic |
| 6# | 4# OR 5# |
| 7# |  |

**Web of Science**

| 1# | TS=(acupuncture OR acupuncture therapy OR Pharmacopuncture OR Acupuncture Treatment OR Acupuncture Treatments OR Pharmacoacupuncture Treatment OR Pharmacoacupuncture Therapy OR Acupotomy OR Acupotomies OR electroacupuncture OR warm needling OR moxibustion OR cupping OR auricular needle OR eye acupuncture OR scalp acupuncture OR body acupuncture OR acupoint OR medicinal vesiculation OR crude herb moxibustion) |
| --- | --- |
| 2# |  |

**CNKI**

( TKA=‘针灸’ + ‘针刺’ + ‘电针’ + ‘温针’ + ‘灸法’ + ‘罐法’ + ‘耳针’ + ‘头皮针’ + ‘体针’ + ‘穴位注射’ + ‘穴位埋线’ + ‘天灸’ + ‘火针’ + ‘针法’) and (TKA=‘痛性糖尿病周围神经病变’ + ‘糖尿病’ + ‘糖尿病疼痛性周围神经’ + ‘糖尿病周围神经’ + ‘糖尿病神经’) and (TKA=‘VAS’ + ‘视觉模拟评分法’ + ‘TCSS’ + ‘SF-36’ + ‘多伦多临床评分系统’ + ‘SF36’ + ‘健康调查简表’)

**CBM**

( "针灸"[常用字段:智能] OR "针刺"[常用字段:智能] OR "电针"[常用字段:智能] OR "温针"[常用字段:智能] OR "灸法"[常用字段:智能] OR "罐法"[常用字段:智能] OR "耳针"[常用字段:智能] OR "头皮针"[常用字段:智能] OR "体针 "[常用字段:智能] OR "穴位注射"[常用字段:智能] OR "穴位埋线"[常用字段:智能] OR "天灸"[常用字段:智能] OR "火针"[常用字段:智能] OR "针法"[常用字段:智能]) AND( "痛性糖尿病周围神经病变"[常用字段:智能] OR "糖尿病"[常用字段:智能] OR "糖尿病疼痛性周围神经"[常用字段:智能] OR "糖尿病周围神经 + 糖尿病神经"[常用字段:智能]) AND( "VAS"[常用字段:智能] OR "视觉模拟评分法"[常用字段:智能] OR "SF"[常用字段:智能] OR "SF36"[常用字段:智能] OR "健康调查简表"[常用字段:智能])

**China Science and Technology Journal Database**

(M="针灸" OR "针刺" OR "电针" OR "温针" OR "灸法" OR "罐法" OR "耳针" OR '头皮针" OR "体针" OR "穴位注射" OR "穴位埋线" OR "天灸" OR "火针" OR "针法")) and (M="痛性糖尿病周围神经病变" OR "糖尿病" OR "糖尿病疼痛性周围神经" OR "糖尿病周围神"经 + "糖尿病神经")) and (M="VAS" OR "视觉模拟评分法" OR "SF" OR "SF36" OR "健康调查简表"))

**WanFang**

主题:(针灸 OR 针刺 OR 电针 OR 温针 OR 灸法 OR 罐法 OR 耳针 OR 头皮针 OR 体针 OR 穴位注射 OR 穴位埋线 OR 天灸 OR 火针 OR 针法) and 主题:(痛性糖尿病周围神经病变 OR 糖尿病 OR 糖尿病疼痛性周围神经 OR 糖尿病周围神经 OR 糖尿病神经) and 主题:(VAS OR 视觉模拟评分法 OR SF OR SF36 OR 健康调查简表)
