# Supplementary material for: Acupuncture for painful diabetic peripheral neuropathy: a systematic review and meta-analysis
Source: Front Neurol. 2023 Nov 16;14:1281485. doi: 10.3389/fneur.2023.1281485 (PMC10690617; doi:10.3389/fneur.2023.1281485)
Supplement: Supplementary file 2 [file Table_2.DOCX]

**Table 2 Characteristics of included studies.**

| **References** | **Country** | **Sample size (E/C)** | **Mean age(Y)(SD)(EG/CG)** | **Course of diabetes [mean(SD)](EG/CG)** | **Experimental treatment** | **Control treatment** | **Location of acupoints** | **Outcome** | **adverse effects** |
| --- | --- | --- | --- | --- | --- | --- | --- | --- | --- |
| Chen, 2015 | China | 174(87/87) | 51.9±7.4 / 52.3±7.9 | 9.4±3.5 / 9.2±3.8(y) | AI(danshen injections) + UC | mecobalamin + UC | limbs | VAS | No side eﬀect  was recorded |
|  |  |  |  |  |  |  |  |  |  |
| Cui et al., 2015 | China | 60(30/30) | 63.20±11.84 / 61.60±8.68 | 15.30±14.94 / 14.90±12.95(m) | A + M + UC | SAA + UC | limbs | SP36-BP | NM |
| Deng and Zhou, 2014 | China | 40(20/20) | 58±6.0 / 60±5.7 | 8.0±6.0 / 8.2±6.3(y) | WN + AI(nervegrowthfactor) + mecobalamin + UC | mecobalamin + UC | limbs | clinical efficacy based on VAS scores | 10% patients in PDPN  ： Transient and  non-persistent pain |
|  |  |  |  |  |  |  |  |  |  |
|  |  |  |  |  |  |  |  |  |  |
| Deng et al., 2021 | China | 60(30/30) | 59±9 / 57±9 | 10.4±3.9 / 9.3±3.6(y) | A + UC | pregabalin + UC | limbs | VAS, SP36-BP | No side eﬀect  was recorded |
|  |  |  |  |  |  |  |  |  |  |
| Garrow et al., 2014 | USA | 45(24/21) | 68±11.1 / 63±10.8 | 12.2±7.4 / 11.0±9.2(y) | A + UC | SA + UC | limbs | VAS, SP36-BP | One chest pain,  one leg pain  in EG; one localized  swelling of leg in CG |
| Hu et al., 2014 | China | 80(40/40) | 55.8±10.06 / 56.6±9.75 | NM | A + WN + gabapentin + UC | gabapentin + UC | head and limbs | clinical efficacy based on VAS scores | NM |
| Kong, 2015 | China | 80(40/40) | NM | NM | A + WN + gabapentin + UC | gabapentin + UC | head and limbs | clinical efficacy based on VAS scores | NM |
| Li et al., 2020 | China | 55(28/27) | 63.07±6.94 / 64.37±5.52 | 11.89±6.90 / 12.63±7.43(y) | M + mecobalamin + UC | mecobalamin + UC | back and limbs | SP36-BP | No side eﬀect  was recorded |
| Li, 2015 | China | 30(15/15) | 72±5.82 (2 groups) | NM | A + mecobalamin + UC | mecobalamin + UC | back, abdomen and limbs | clinical efficacy based on VAS scores | NM |
| Liu and He, 2019 | China | 100(50/50) | 55.86±1.14 / 55.74±1.10 | NM | EA + UC | UC | back, abdomen and limbs | VAS | No side eﬀect  was recorded |
| Liu et al., 2020(a) | China | 60(30/30) | 61.36±3.08 / 61.89±3.57 | 6.98±1.85 / 7.68±1.24(y) | A + lipoic acid + UC | lipoic acid + UC | limbs | VAS | NM |
| Liu et al., 2020(b) | China | 62(31/31) | 66.77±6.43 / 65.48±8.09 | 10.92±6.82 / 9.82±6.11(y) | A+WN + UC | mecobalamin + UC | limbs | SP36-BP, TCSS | NM |
| Liu et al., 2022(a) | China | 110(55/55) | 56.1±12.69 / 57.75±9.62 | 10.32±8.2 / 9.88±6.9(y) | AI(raceanisodamine) + epalrestat + lipoic acid + UC | epalrestat + lipoic acid + UC | limbs | VAS, clinical efficacy based on VAS scores | NM |
| Liu et al., 2022(b) | China | 150(75/75) | 60.23±7.54 / 59.48±7.63 | 6.37±1.24 / 6.56±1.14(y) | A + mecobalamin + UC | mecobalamin + UC | limbs | VAS, TCSS | No side eﬀect  was recorded |
| Peng, 2016 | China | 86(43/43) | 56.39±4.63 / 57.52±5.26 | 1.15±0.41 / 1.23±0.32(y) | A + gliclazide + mecobalamin | gliclazide + mecobalamin | back, abdomen and limbs | VAS | No side eﬀect  was recorded |
| Shu et al., 2021 | China | 60(30/30) | 64±8 / 65±7 | 12.50（4.75，22.00） / 19.50（11.75，20.00） [M（QU，QL）] | A + mecobalamin + UC | mecobalamin + UC | back and limbs | VAS, TCSS | NM |
| Song, 2018 | China | 80(40/40) | 62.54±3.52 / 62.58±3.26 | 4.29±0.83 / 4.12±0.76(y) | A + mecobalamin + UC | mecobalamin + UC | back and limbs | VAS | NM |
| Tang et al., 2018 | China | 145(70/75) | 61 / 56 | 13 / 11(y) | M + mecobalamin + UC | mecobalamin + UC | limbs | VAS | No side eﬀect  was recorded |
|  |  |  |  |  |  |  |  |  |  |
| Tian and Liu, 2019 | China | 80(40/40) | 62.66±3.41 / 62.38±3.13 | 4.35±0.81 / 4.09±1.12(y) | A + mecobalamin + UC | mecobalamin + UC | back, abdomen and limbs | VAS, SP36-BP, TCSS | NM |
| Wang et al., 2021 | China | 138(69/69) | 61.3±7.4 / 62.1±8.3 | 10.4±4.2 / 10.9±4.9(y) | EA + α-lipoic + acidepalrestat + UC | α-lipoic + acidepalrestat + UC | back and limbs | VAS, TCSS | No side eﬀect  was recorded |
| Wang, 2018 | China | 100(50/50) | 62.6±5.8 / 62.7±5.3 | 4.7±1.1 / 4.7±1.3(y) | A + M + UC | UC | limbs | SP36-BP | NM |
| Wu et al., 2018 | China | 88(44/44) | 62.58±3.57 / 62.59±3.58 | 4.28±0.88 / 4.29±0.84(y) | A + mecobalamin | mecobalamin | back and limbs | VAS, TCSS | NM |
| Yang and Kong, 2019 | China | 96(48/48) | NM | 15.2±2.2 / 14.3±2.8(m) | A + massage | mecobalamin + vitamin B1 | NM | VAS | NM |
| Zheng, 2017 | China | 120(60/60) | 56.19±7.31 / 56.27±7.23 | 12.53±3.62 / 12.46±3.74(y) | M | mecobalamin | NM | VAS, SP36-BP | NM |
| Zhu et al., 2016 | China | 56(28/28) | 65 / 63 | 16 / 15(y) | A | mecobalamin | limbs | clinical efficacy based on VAS scores | NM |

Y, years old; SD, standard deviation; EG, experimental group; CG, control group; A, acupuncture; EA, electroacupuncture; AI,acupoint injection; WN, warm needing; M, moxibustion; SA, sham acupuncture; SAA, sham auricular acupuncture; UC, usual care; NM, not mention; y, year(s); m, month(s).
