# Supplementary material for: Acupuncture for painful diabetic peripheral neuropathy: a systematic review and meta-analysis
Source: Front Neurol. 2023 Nov 16;14:1281485. doi: 10.3389/fneur.2023.1281485 (PMC10690617; doi:10.3389/fneur.2023.1281485)
Supplement: Supplementary file 3 [file Table_3.docx]

**Table 3 GRADE classification for effect of acupuncture in pain intensity in patients with**

**painful diabetic peripheral neuropathy.**

| **Certainty assessment** | | | | | | | **№ of patients** | | **Effect** | | **Certainty** | **Importance** |
| --- | --- | --- | --- | --- | --- | --- | --- | --- | --- | --- | --- | --- |
| **№ of studies** | **Study**  **design** | **Risk of bias** | **Inconsistency** | **Indirectness** | **Imprecision** | **Other**  **considerations** | **acupuncture**  **with routine**  **treatment** | **routine**  **treatment** | **Relative (95% CI)** | **Absolute (95% CI)** |  |  |

**VAS**

| 16 | randomised trials | seriousa | seriousb | not serious | not serious | none | 773 | 779 | - | MD **1.62 lower**  (2 .01  lower to  1 .23  lower) | ⨁⨁◯◯ Low | CRITICAL |
| --- | --- | --- | --- | --- | --- | --- | --- | --- | --- | --- | --- | --- |

**SF- 36 Bodily Pain**

| 8 | randomised trials | seriousc | seriousb | not serious | not serious | none | 293 | 289 | - | S MD  **2.44**  **higher**  (1 .33  higher to  3 .56  higher) | ⨁⨁◯◯ Low | CRITICAL |
| --- | --- | --- | --- | --- | --- | --- | --- | --- | --- | --- | --- | --- |

**TCSS**

| 6 | randomised trials | seriousc | not serious | not serious | not serious | none | 288 | 289 | - | MD **1.42 lower**  (1 .65  lower to  1 .19  lower) | ⨁⨁⨁◯  Moderate | IMPORTANT |
| --- | --- | --- | --- | --- | --- | --- | --- | --- | --- | --- | --- | --- |

**Effective Rate**

| 6 | randomised trials | seriousc | not serious | not serious | not serious | none | 158/198  (79 .8%) | 114/198 (57 .6%) | **RR 1.39**  (1 .21 to 1 .59) | **225**  **more per 1,000**  (from  121  more to  340  more) | ⨁⨁⨁◯  Moderate | CRITICAL |
| --- | --- | --- | --- | --- | --- | --- | --- | --- | --- | --- | --- | --- |

**CI:** confidence interval; **MD:** mean difference; **RR:** risk ratio; **SMD:** standardised mean difference

**Explanations**

a . Publication bias detected by funnel plots .

b. The I² —whic h quantifies the proportion of the variation in point estimates due to among-study differences--is large

c . Blind deletion
